# Changes in the profile properties and chemical weathering characteristics of cultivated soils affected by anthropic activities

**DOI:** 10.1038/s41598-021-00302-w

**Published:** 2021-10-21

**Authors:** Jiangwen Li, Jing Du, Shouqin Zhong, En Ci, Chaofu Wei

**Affiliations:** 1 College of Resources and Environment/Key Laboratory of Eco-Environments in Three Gorges Region (Ministry of Education), Southwest University, No.2 Tiansheng Road, BeiBei District, Chongqing, 400715 People’s Republic of China; 2 Chongqing Agricultural Ecology and Resources Protection Station, Chongqing, 401121 People’s Republic of China; 3 Key Laboratory of Arable Land Conservation (Southwestern China), Ministry of Agriculture, Chongqing, 400715 People’s Republic of China; 4 State Cultivation Base of Eco-Agriculture for Southwest Mountainous Land, Southwest University, Chongqing, 400715 People’s Republic of China

**Keywords:** Solid Earth sciences, Engineering

## Abstract

The study of the pedogenic process in response to natural evolution, gradual anthropogenic shifts and engineering upheavals is of great significance for understanding, utilizing and transforming nature in the future. Although scholars have considered anthropic activities to be an important factor affecting pedogenesis, research on how and how much anthropic activities influence the soil-forming process is scant. This paper was conducted to analyse pedogenic characteristics dominated by anthropic activities. In this study, the parent materials and soils undergoing natural evolution (NE), tillage perturbation (TP) and engineering perturbation (EP) were selected as research objects. The genetic characteristics of soils undergoing NE, TP and EP are investigated mainly from three aspects: soil profile macromorphological characteristics, soil physical and chemical properties and chemical weathering characteristics. The results indicated that the influence of anthropic activities (TP and EP) on the process of pedogenesis is complicated. First, compared with NE, TP decreases the thickness of topsoil from 22.2 to 21.2 cm, while EP increases the thickness of topsoil from 22.2 to 23.2 cm, and EP causes the soil to have a high profile development index. Second, compared with TP, EP can improve bulk density (BD), soil organic carbon (SOC), total nitrogen (TN) and cation exchange capacity (CEC), Finally, the chemical weathering intensity differed among NE, TP and EP and followed the order of TP > NE > EP. Therefore, in the future, the genetic characteristics of soils dominated by anthropic activities should be considered. This will help us systematically understand the genesis and evolutionary characteristics of soil and lay a foundation for further perfecting the diagnostic horizon and diagnostic characteristics of the Soil Taxonomy and World Reference Base.

## Introduction

Soil, as a natural body, has its own unique patterns of genesis and development, similar to other natural bodies. In long-term studies, parent materials, climate, topography, biology and time are regarded as the five major factors affecting soil formation^1^. Due to anthropic pressure, anthropic activities have been gradually regarded as a sixth soil-forming factor^2–4^. Generally, humans change soil properties and pedogenetic processes by land preparation, terrace construction, fertilization, irrigation and other anthropic activities, thereby speeding up the rate of soil formation^5–7^. However, acidification, salinization, pollution, compaction, disturbance, erosion and other problems caused by anthropic activities can cause serious damage to the soil^8–11^. Therefore, the influence of human activities on soil formation and evolution is very complicated. If effective artificial measures are adopted to achieve the purpose of sustainable scientific utilization and protection of soil, it is of great significance to study the impact of anthropic activities on the genesis and evolution of soil.

Pedogenesis refers to the evolution from the profile scale to the regional scale, which includes significant changes in soil under physical, chemical or biological conditions^4, 12^. Generally, soil formation is relatively slow and follows centuries to billions of years of natural evolution^5^. This process of evolution tends towards the directional development of mature soil bodies to ensure their balance with the material and energy in the environment^13^. However, due to the participation of anthropic activities, the quasi-steady state of soil processes has been fundamentally destroyed^14^. For example, anthropic activities mainly narrow the range of soil development to only one function (e.g., crop production for agropedogenesis) and attach importance to only one function, thereby leading to soil degradation^9^. Although anthropic activities narrow the range of soil development to only one function, the effect of anthropic activities on soil formation cannot be ignored in intensity and rate.

In the intensification of agriculture, a series of anthropic activities may accelerate the rate of pedoturbation and thereby change the formation process and evolution of the soil^3, 4, 15, 16^. The influence of anthropic activities on pedogenesis and evolution can be summarized as follows: (1) Changes in the macromorphological characteristics of a soil profile. Activities such as topsoil stripping and backfilling, sloping terrace construction and field ridge construction can strongly disturb the soil by changing the thickness and configuration of the soil layer^16, 17^. (2) Changes in soil-forming speed. Compared with natural evolution, anthropic activities can accelerate soil physical weathering and thereby increase pedogenetic capacity^7^. Tang et al.^5^ showed that farming plot reconstruction engineering accelerates soil physical weathering while decreasing chemical weathering. (3) Changes in the direction of material movement. Periodic artificial flooding and drainage lead to the coupled processes of oxidation—reduction and leaching—deposition of iron during the evolution of paddy soil^18^.

During the long-term relationship between humans and soil, the characteristics and behaviour of soil have been significantly influenced and changed by anthropic activities and tools for a long time^10, 19^, and anthropic activities are regarded as a combination of factors affecting a series of processes rather than as a single factor affecting the soil-forming process^20, 21^. In fact, the World Reference Base (WRB) established the Anthrosol and Technosol reference soil groups (RSGs) and formulated corresponding evaluation indicators^22^. Scholars have realized that anthropic activities are an important factor affecting pedogenesis, but research on how and how much anthropic activities influence the soil-forming process is scant. With the rapid development of agriculture, society and the economy, farming plot reconstruction engineering is commonly applied to farmland to diminish the obstacles of cultivated land fragmentation and scale problems in the process of agricultural intensification. Therefore, it is necessary for us to study the influence of farming plot reconstruction engineering on soil genetic characteristics.

The objectives of this study were (1) to analyse the influence of anthropic activities on pedogenesis; (2) to study the soil genetic characteristics of engineering perturbations; and (3) to highlight the research of soil genetic classification considering the anthropic activities.

## Materials and methods

### Study area

**Figure 1:**
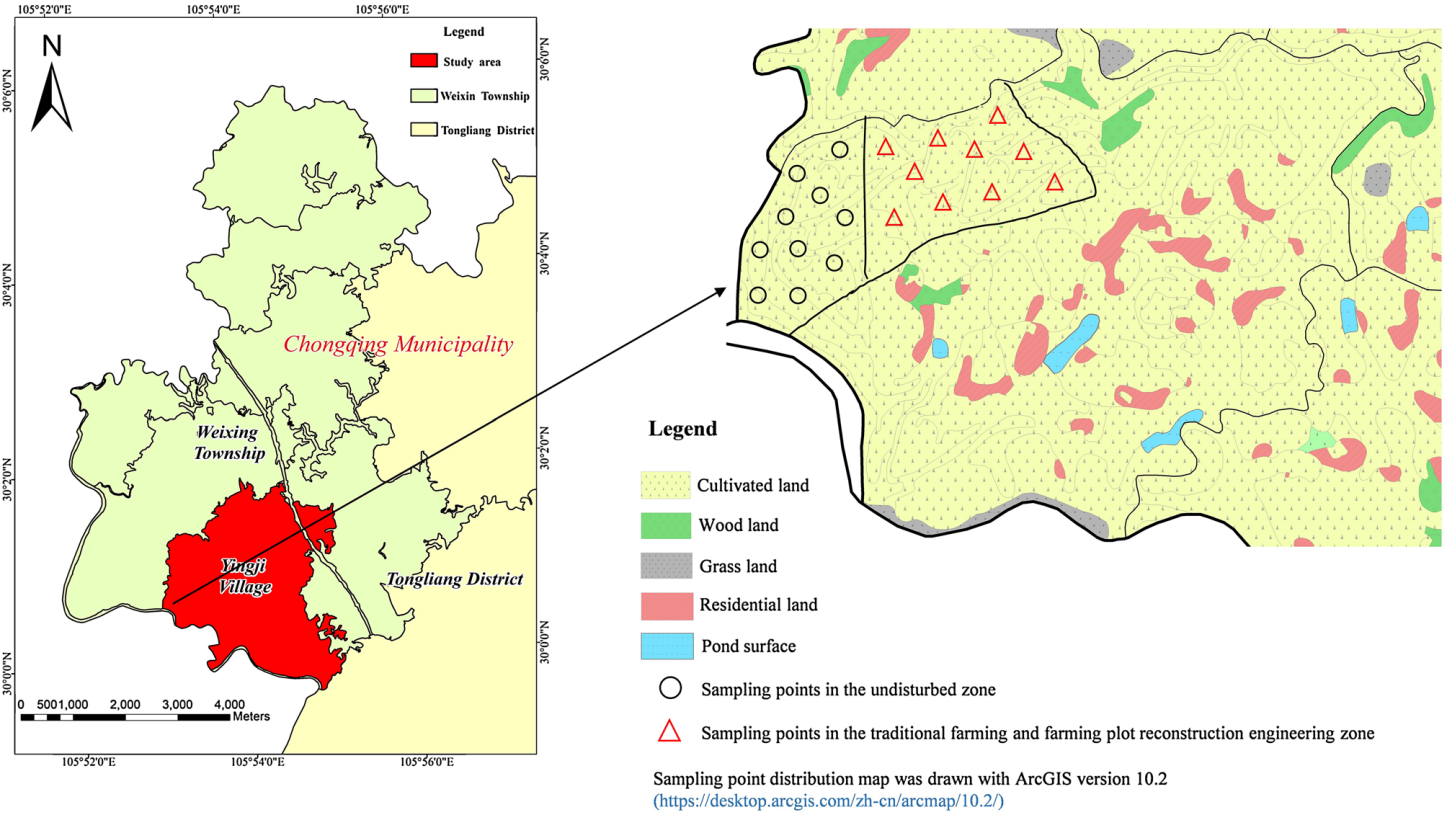
Study area.

Yingji village is located in north-western Chongqing, China (Fig. 1). This area is mainly characterized by low-slope hilly topography and a subtropical monsoon climate, with a mean annual temperature of 18.1 °C. The average annual precipitation is 1071 mm, which is concentrated in May and September. The parent rocks of the study are mainly include sandstone and mudstone, most of which are Jurassic semiarid lacustrine sedimentary rocks. To meet the needs of the local government to improve agricultural production capacity and optimize land structure, farming plot reconstruction engineering was implemented in Yingji village. In this area, farming plot reconstruction engineering involved topsoil stripping and backfilling, sloping terrace construction and field ridge construction. The design requirements of the specific engineering are consistent with those of Tang et al.^5^.

### Sample collection

**Figure 2:**
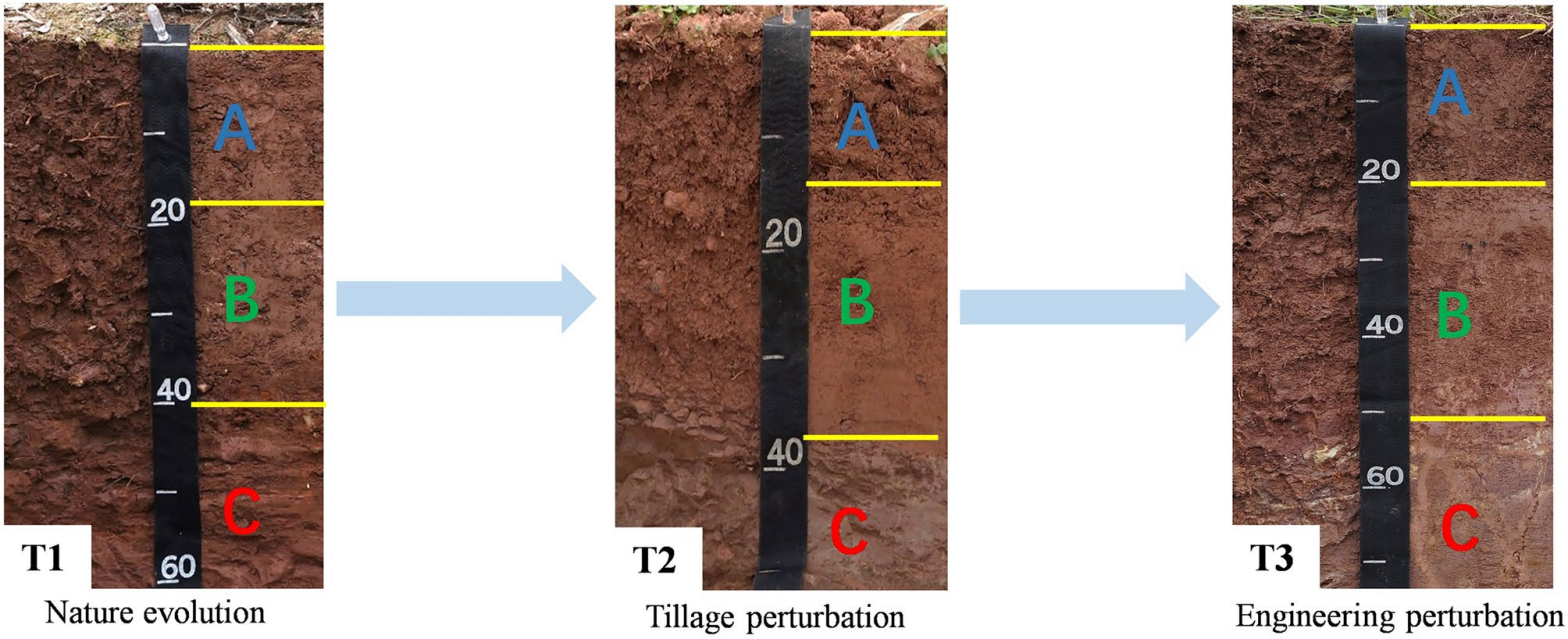
Soil profiles in different pedogenic processes.

The soil samples were classified as Inceptisol or Entisols according to the United States Department of Agriculture (USDA) taxonomy. To study the influence of natural evolution (NE) and anthropic activities (tillage perturbation (TP) and engineering perturbation (EP)) on the soil-forming process, three types of samples were collected, and ten soil profiles were excavated for each type of sample. The first type of sample (T1) was collected in the undisturbed zone, and this zone was selected around the farming plot reconstruction engineering; the second type of sample (T2) was collected in the traditional farming zone, namely, the zone before farming plot reconstruction engineering; and the third type of sample (T3) was collected in the farming plot reconstruction engineering zone, namely, the zone after farming plot reconstruction engineering (Figs. 1, 2). We divided the soil profile horizon, recorded the corresponding morphological characteristics, and collected samples at each horizon according to the *Field Book for Describing and Sampling Soils Version 3.0*^23^. Approximately 3 kg soil/rock samples were collected according to the sequence from the parent rock (C horizon) to the topsoil layer (A horizon). Finally, gravel, plant roots, animal residues, and other impurities were removed, and all samples were ground (2 mm) and then stored in airtight bags for further laboratory analysis.

### Sample testing and data analysis

The bulk density (BD) was measured by the cutting ring method; soil density was measured by the density bottle method; pH was measured (soil:water = 1:2.5) by the SX836 Model; soil organic carbon (SOC) was measured using a potassium dichromate oxidation method; cation exchange capacity (CEC) was determined by NaOAc-EDTA; total nitrogen (TN) was measured by the Kjeldahl method; soil particle composition (SPC) was measured by sieving and sedimentation processes and a pipette method; and porosity was calculated by using BD and soil density^24^. The geochemical elements (Si, Al, Fe, Ti, Mn, Ca, Mg, K, Na, and P) were measured using X-ray fluorescence spectrometry. The combination of various elements was selected to form the judgement indicator of rock/soil weathering. The weathering index was the basis of the quantitative evaluation of mineral weathering intensity, which is usually designed to measure the loss of mobile elements (e.g., K, Na, Ca, and Mg) relative to immobile elements (e.g., Ti). In this study, several weathering indices were selected to evaluate the weathering characteristics of soils under natural evolution or anthropic activities.

The chemical index of alteration (CIA) is an important index to judge the chemical weathering intensity of sediments, which can effectively indicate the weathering intensity of feldspar transformation to clay minerals in samples. Generally, the chemical weathering intensity is divided into three phases: a CIA value of 50–65 represents a weak weathering intensity, a value of 65–85 represents a moderate chemical weathering intensity, and a value of 85–100 represents a strong weathering intensity^25^. The CIA can be calculated with Eq. (1)^26^:

1 $${\text{CIA}} = \{ {\text{Al}}_{{2}} {\text{O}}_{{3}} {/}\left( {{\text{Al}}_{{2}} {\text{O}}_{{3}} + {\text{CaO}}^* + {\text{Na}}_{{2}} {\text{O}} + {\text{K}}_{{2}} {\text{O}}} \right)\} \times 100$$

where all the macroelement oxides are expressed by molar fraction. CaO* represents the molar fraction of CaO in silicate minerals and is calculated by the method of McLennan^27^.

The weathering leaching coefficient (WLC) reflects the intensity of soil weathering and leaching^28^. The WLC can be calculated with Eq. (2):

2 $${\text{WLC}} = \frac{{\left( {\text{K}}_2{\text{O}} + {\text{Na}}_2{\text{O}} + {\text{CaO}}^* + {\text{MgO}} \right)}}{{{\text{Al}}_2{\text{O}}_3}}$$

The plagioclase index of alteration (PIA) is used to monitor the intensity of plagioclase weathering alone^29^. The PIA can be calculated with Eq. (3):

3 $${\text{PIA}} = \left( {\frac{{{\text{Al}}_2{\text{O}}_3 - {\text{K}}_2{\text{O}}}}{{{\text{Al}}_2{\text{O}}3 + {\text{CaO}}^* + {\text{Na}}_2{\text{O}} - {\text{K}}_2{\text{O}}}}} \right) \times 100$$

The profile development index (PDI) is used to reflect the soil-forming environment. The selection and assignment of each description feature reference the method of Harden^26^, as shown in Appendix A.

All statistical analyses were performed with SPSS 19.0. Linear correlation analysis was conducted to research the relationship between weathering indices. A sampling point distribution map was drawn with ArcGIS 10.2, and other maps were created with Origin 2019.

## Results

### Soil profile macromorphological characteristics

**Figure 3:**
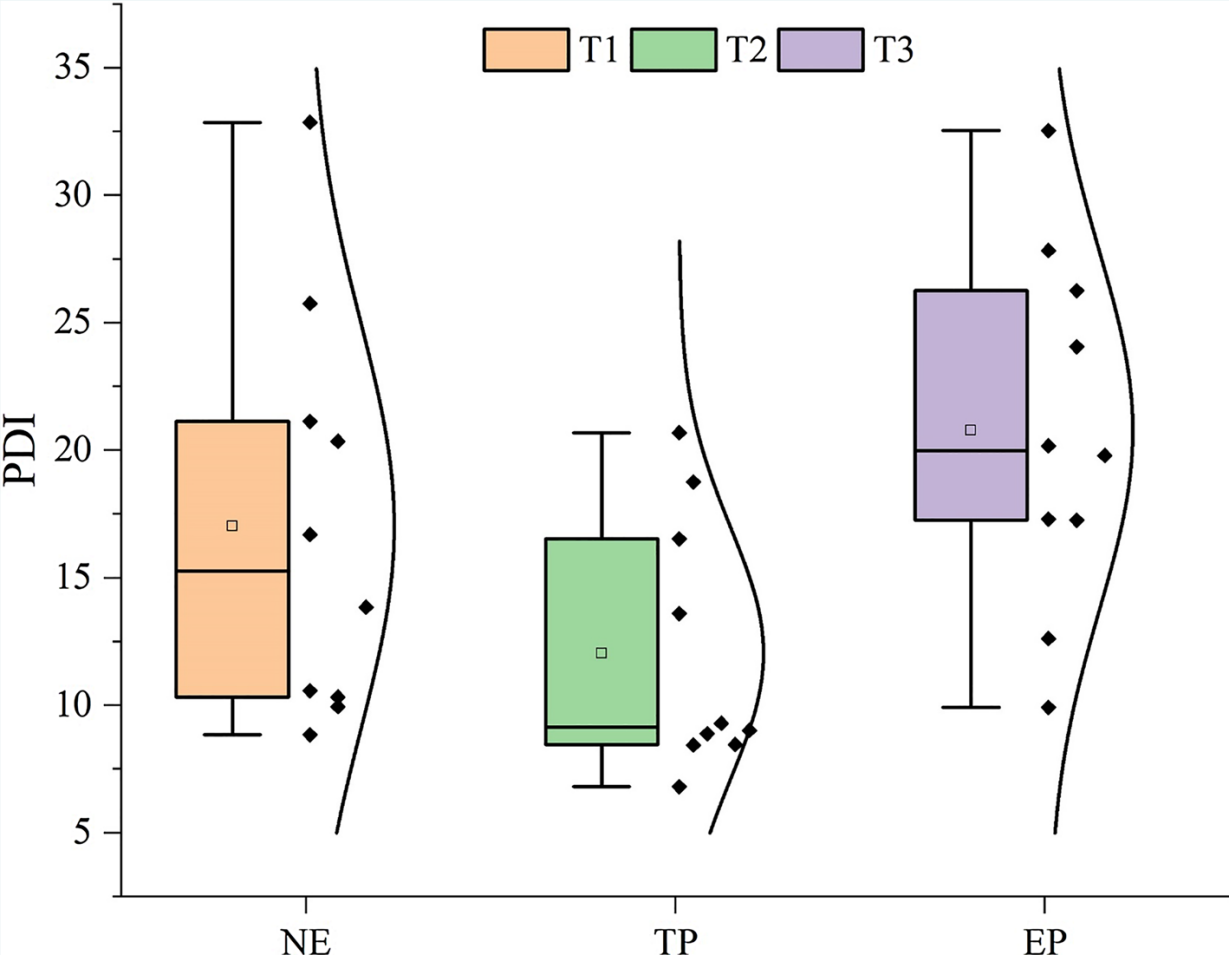
PDI distribution characteristics of profiles in different pedogenic processes. PDI = profile development index; T1, T2 and T3 represent the soils undergoing natural evolution, tillage perturbation and engineering perturbation, respectively.

Soil morphology descriptions are presented in the supplementary data (Appendix B) and Table 1, and soil thickness, structure and textures are mainly mentioned here. In T1, the soil profile (S1–S10) mainly involved A-B-C and A-C patterns. The thickness of the soil layer ranged from 18 to 59 cm, among which the average topsoil (A horizon) thickness was 22.20 cm, and its coefficient of variation (CV) was 21.85%. The soil structure mainly included granular and angular blocky, and the soil texture mainly included loamy clay and clay. In T2, the soil profile (P1–P10) patterns were simple, and most of them were A–B–C and A–C patterns. The thickness of the soil layer ranged from 15 to 55 cm, and the average A horizon thickness was 21.20 cm, with a CV of 26.65%. The soil structure was mainly granular and angular blocky, and the soil texture mainly included sandy loam and clay loam. In T3, most of the ten profiles (P′1–P′10) mainly involved A-B-C patterns. The thickness of the soil layer ranged from 30 to 60 cm, and the average A horizon thickness was 24.2 cm, with a CV of 15.56%. The soil structure was mainly granular and angular blocky, and the soil texture mainly included clay loam. This indicates that anthropic activities mainly change the thicknesses and soil texture of the soil layer. Based on this, the PDI was used to characterize the macromorphological characteristics of the soil profile and shows the order of T3 > T1 > T2 (Fig. 3). This shows that engineering perturbation gives the soil a high profile development degree.

**Table 1:** Physical properties of soil profiles in different pedogenic processes.

Sample types	Profile horizon	Depth (cm)	BD (g·cm^−3^)	Porosity (%)	SPC (%)		
					2–0.02 mm	0.02–0.002 mm	< 0.002 mm
T1	A horizon (mean ± SD) (n = 10)	22.20 ± 4.85	1.34 ± 0.12	45.53 ± 4.65	35.65 ± 15.43	29.77 ± 12.10	34.58 ± 10.14
	B horizon (mean ± SD) (n = 5)	20.20 ± 8.61	1.58 ± 0.14	38.35 ± 5.33	34.82 ± 16.75	30.32 ± 10.48	34.86 ± 13.98
	C horizon (mean ± SD) (n = 10)	–	–	–	36.43 ± 15.85	30.54 ± 11.36	33.03 ± 9.81
T2	A horizon (mean ± SD) (n = 10)	21.20 ± 5.65	1.26 ± 0.13	49.99 ± 5.05	58.95 ± 16.84	23.11 ± 9.24	17.94 ± 8.07
	B horizon (mean ± SD) (n = 3)	26.00 ± 12.17	1.47 ± 0.06	42.80 ± 2.63	60.13 ± 19.17	28.16 ± 15.11	11.71 ± 4.52
	C horizon (mean ± SD) (n = 10)	–	–	–	76.83 ± 16.42	14.41 ± 10.23	8.76 ± 6.62
T3	A horizon (mean ± SD) (n = 10)	23.20 ± 3.36	1.38 ± 0.12	43.84 ± 4.60	50.48 ± 7.82	27.21 ± 4.49	22.41 ± 4.52
	B horizon (mean ± SD) (n = 8)	32.00 ± 15.00	1.59 ± 0.09	37.77 ± 3.71	60.87 ± 3.50	23.76 ± 2.23	15.37 ± 2.05
	C horizon (mean ± SD) (n = 10)	–	–	–	74.91 ± 13.02	14.32 ± 7.84	10.77 ± 6.23

T1, T2 and T3 represent the soils undergoing natural evolution (NE), tillage perturbation (TP) and engineering perturbation (EP), respectively; BD = bulk density; SPC = soil particle composition; SD = standard deviation; n represents sample size.

### Soil physical and chemical characteristics

Soil physical properties (bulk density, porosity and soil particle composition) are important indicators reflecting soil permeability and nutrient supply capability. From the A to B horizon, the BD ranges from 1.34 to 1.58 g cm^−3^, from 1.26 to 1.47 g cm^−3^, and from 1.38 to 1.59 g cm^−3^; the porosity ranged from 45.53 to 38.35%, from 49.99 to 42.80%, and from 43.84 to 37.77% (Table 1). This indicates that the increase in soil depth led to an increase in BD and a decrease in porosity. For topsoil (A horizon), compared with NE, TP reduced BD, clay and silt content and increased soil porosity and sand content; EP reduced clay and silt content and increased sand content. However, anthropic activities (TP and EP) can effectively improve the uniformity of topsoil particles. For sand, the CVs of T2 and T3 were 14.66% and 27.79% lower than those of T1, respectively. For silt, the CVs of T2 and T3 were 0.66% and 24.14% lower than those of T1, respectively. For clay, the CV of T3 was 9.15% lower than that of T1, respectively.

**Figure 4:**
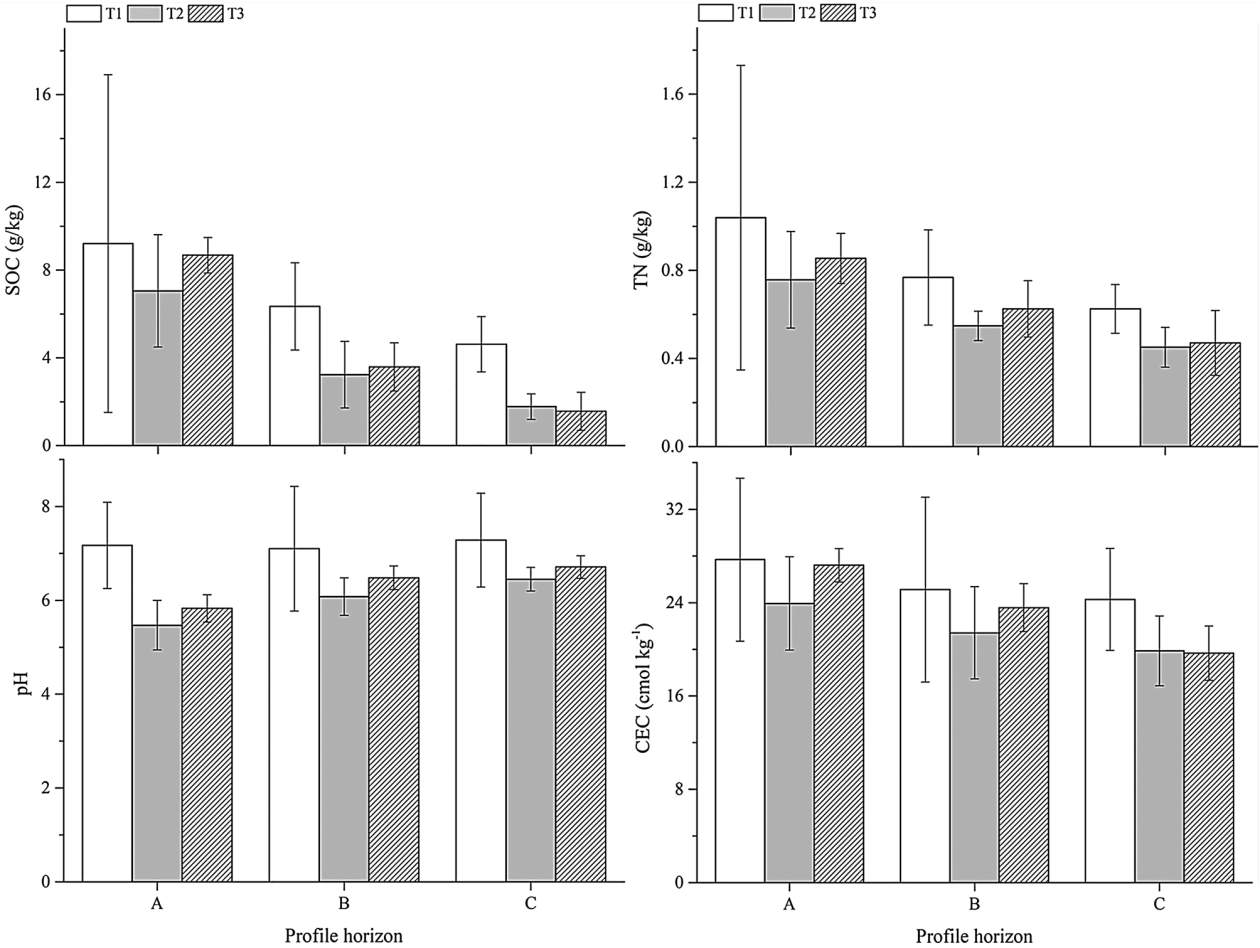
The change in chemical properties for soil horizons in different pedogenic processes. SOC = soil organic carbon; TN = total nitrogen; CEC = cation exchange capacity.

Soil chemical properties are important indicators reflecting soil fertility. From the A to C horizon, the SOC content affected by NE, TP and EP decreased from 9.21, 7.04 and 8.68 to 4.62 g/kg, 1.78 g/kg and 1.56 g/kg; the TN content decreased from 1.04, 0.76 and 0.85 to 0.63 g/kg, 0.45 g/kg and 0.47 g/kg; the CEC decreased from 27.69, 23.93 and 27.20 to 24.28 g/kg, 19.87 g/kg and 19.66 g/kg; and the pH increased from 7.17, 5.47 and 5.83 to 7.28, 6.45 and 6.71, respectively (Fig. 4). Furthermore, there are also differences in the effects of natural evolution and anthropic activities on the chemical properties of topsoil. The SOC of T1 is 23.45% and 5.75% higher than that of T2 and T3; the TN of T1 is 26.92% and 18.27% higher than that of T2 and T3; the pH of T1 is higher than that of T2 and T3; the CEC of T1 was 13.58% and 1.77% higher than that of T2 and T3 (Fig. 4). This shows that anthropic activities reduced the soil fertility level, but after engineering perturbation, the soil fertility level was improved. Meanwhile, anthropic activities can decrease the CV of chemical properties, and the CV of SOC followed the order of T2 (36.31%) > T1 (16.40%) > T3 (9.33%); the CV of TN followed the order of T1 (66.35%) > T2 (28.95%) > T3 (12.94%); the CV of pH followed the order of T1 > T2 > T3. The CV of CEC followed the order of T1 (25.21%) > T2 (16.72%) > T3 (5.22%) (Fig. 4).

### Geochemical weathering characteristics

**Figure 5:**
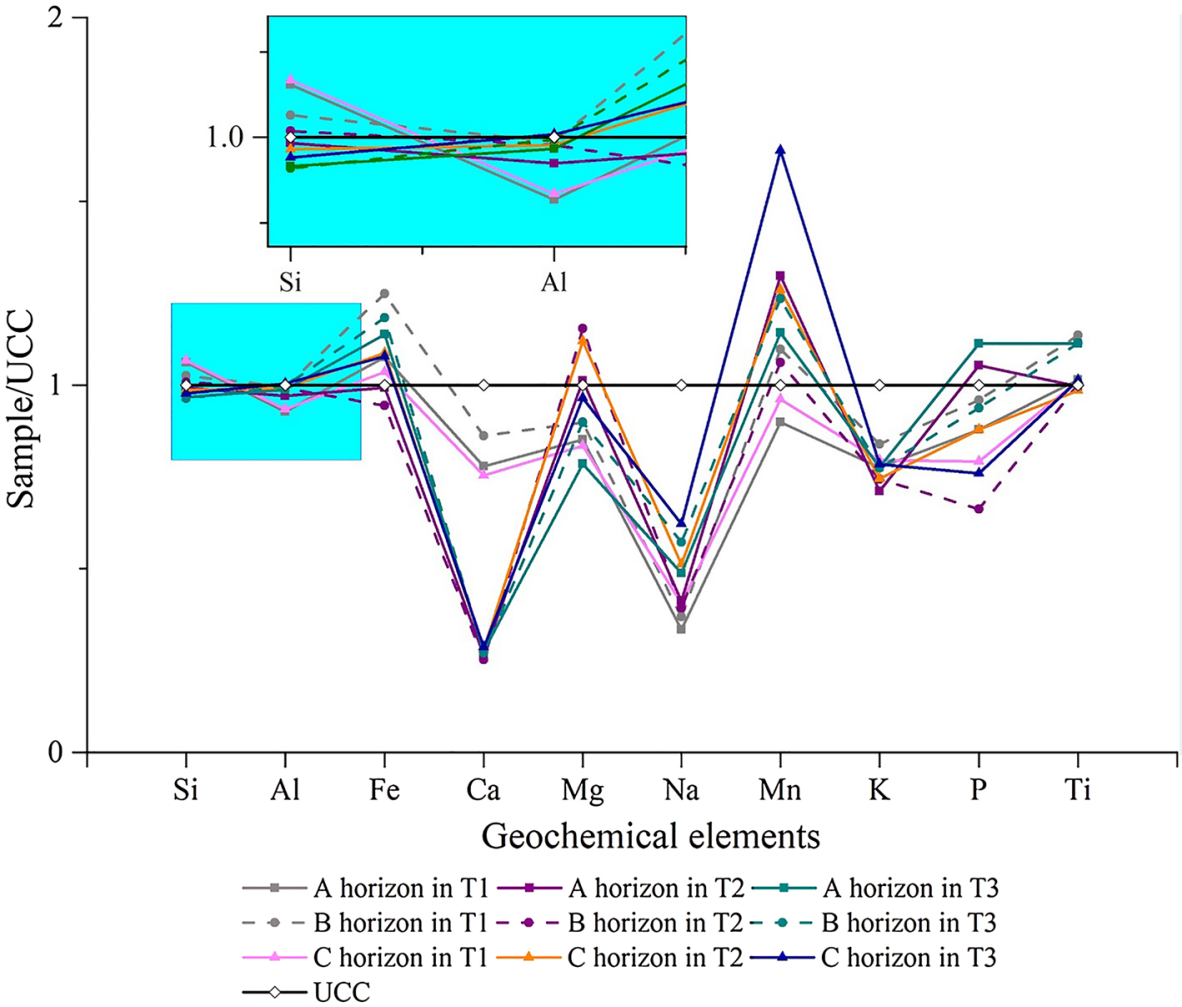
Patterns of UCC-normalized geochemical elements in soil profiles undergoing different pedogenic processes.

The geochemical compositions of T1, T2 and T3 are listed in Table 2, which shows that SiO_2_, Al_2_O_3_ and Fe_2_O_3_ are the most abundant geochemical elements in the soil profile. Compared with the geochemical composition of the upper continental crust (UCC)^30^, the Si, Al and Ti contents are relatively stable, with high Fe and Mn contents and low Ca, Na and K contents (Fig. 5). From the deviation degree between the curves of the soil and the UCC, it can be seen that the loss of K and Ca is relatively strong in T2 and T3, while the loss of Na is relatively strong in T1.

**Table 2:** Geochemical compositions and weathering index under different pedogenic processes.

Sample types	Horizon	SiO_2_ (%)	Al_2_O_3_ (%)	Fe_2_O_3_ (%)	CaO (%)	MgO (%)	Na_2_O (%)	MnO (%)	K_2_O (%)	P_2_O_5_ (%)	TiO_2_ (%)	CIA	WLC	PIA
T1	A (n = 10)	70.09 ± 6.71	14.09 ± 2.23	5.38 ± 1.30	3.27 ± 3.47	1.88 ± 0.75	1.30 ± 0.56	0.07 ± 0.04	2.64 ± 0.74	0.13 ± 0.05	0.66 ± 0.13	67.35 ± 8.13	0.83 ± 0.20	73.28 ± 9.54
	B (n = 5)	67.70 ± 5.31	15.12 ± 0.78	6.24 ± 0.55	3.62 ± 3.71	1.98 ± 0.51	1.44 ± 0.84	0.09 ± 0.04	2.86 ± 0.72	0.14 ± 0.05	0.74 ± 0.11	67.97 ± 9.90	0.82 ± 0.22	74.44 ± 12.53
	C (n = 10)	70.42 ± 7.32	14.20 ± 2.62	5.18 ± 1.53	3.17 ± 3.00	1.84 ± 0.76	1.58 ± 0.62	0.08 ± 0.03	2.70 ± 0.68	0.12 ± 0.05	0.66 ± 0.16	64.81 ± 6.99	0.87 ± 0.20	70.08 ± 9.42
T2	A (n = 10)	65.55 ± 2.73	14.74 ± 0.34	4.96 ± 0.87	1.11 ± 0.26	2.23 ± 0.43	1.61 ± 0.42	0.10 ± 0.03	2.42 ± 0.24	0.16 ± 0.06	0.65 ± 0.12	68.35 ± 3.25	0.85 ± 0.07	74.22 ± 4.20
	B (n = 3)	66.50 ± 1.90	15.06 ± 0.78	4.72 ± 1.13	1.06 ± 0.13	2.54 ± 0.40	1.53 ± 0.48	0.08 ± 0.03	2.53 ± 0.22	0.10 ± 0.06	0.65 ± 0.07	68.64 ± 2.55	0.89 ± 0.04	74.80 ± 3.33
	C (n = 10)	65.10 ± 2.45	15.07 ± 0.56	5.44 ± 1.09	1.18 ± 0.22	2.46 ± 0.33	1.99 ± 0.21	0.10 ± 0.03	2.53 ± 0.29	0.13 ± 0.04	0.64 ± 0.11	65.78 ± 1.83	0.93 ± 0.08	70.75 ± 2.49
T3	A (n = 10)	63.78 ± 1.24	15.00 ± 0.40	5.69 ± 0.45	1.16 ± 0.09	1.73 ± 0.18	1.90 ± 0.32	0.09 ± 0.02	2.64 ± 0.23	0.17 ± 0.05	0.72 ± 0.03	66.12 ± 1.64	0.80 ± 0.06	71.55 ± 2.18
	B (n = 8)	63.61 ± 1.23	15.16 ± 0.37	5.92 ± 0.52	1.14 ± 0.15	1.98 ± 0.06	2.23 ± 0.23	0.10 ± 0.02	2.63 ± 0.17	0.14 ± 0.03	0.72 ± 0.03	64.77 ± 0.82	0.87 ± 0.02	69.53 ± 1.16
	C (n = 10)	64.44 ± 1.64	15.25 ± 0.28	5.39 ± 0.87	1.20 ± 0.15	2.12 ± 0.22	2.42 ± 0.36	0.13 ± 0.05	2.67 ± 0.18	0.11 ± 0.02	0.66 ± 0.12	63.49 ± 1.62	0.93 ± 0.07	67.78 ± 2.37

T1, T2 and T3 represent the soils undergoing natural evolution (NE), tillage perturbation (TP) and engineering perturbation (EP); CIA = chemical index of alteration; WLC = weathering leaching coefficient; PIA = plagioclase index of alteration.

**Figure 6:**
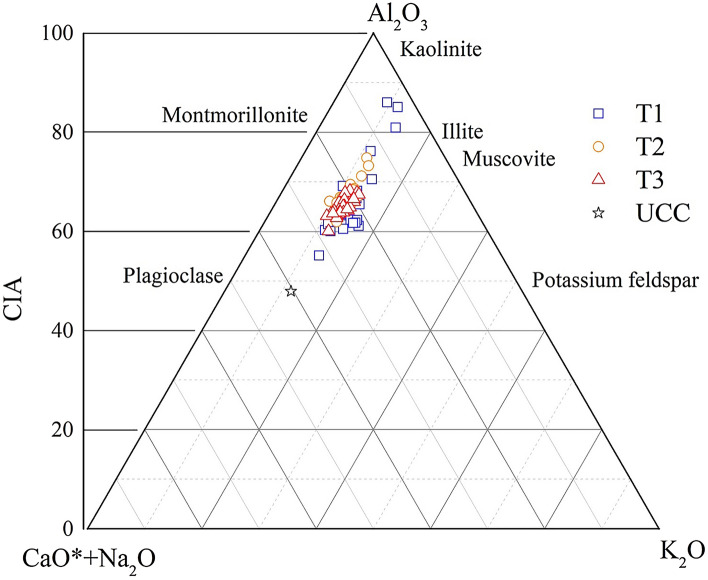
A-CN-K ternary diagram of soil profiles undergoing different pedogenic processes. CIA = chemical index of alteration.

**Figure 7:**
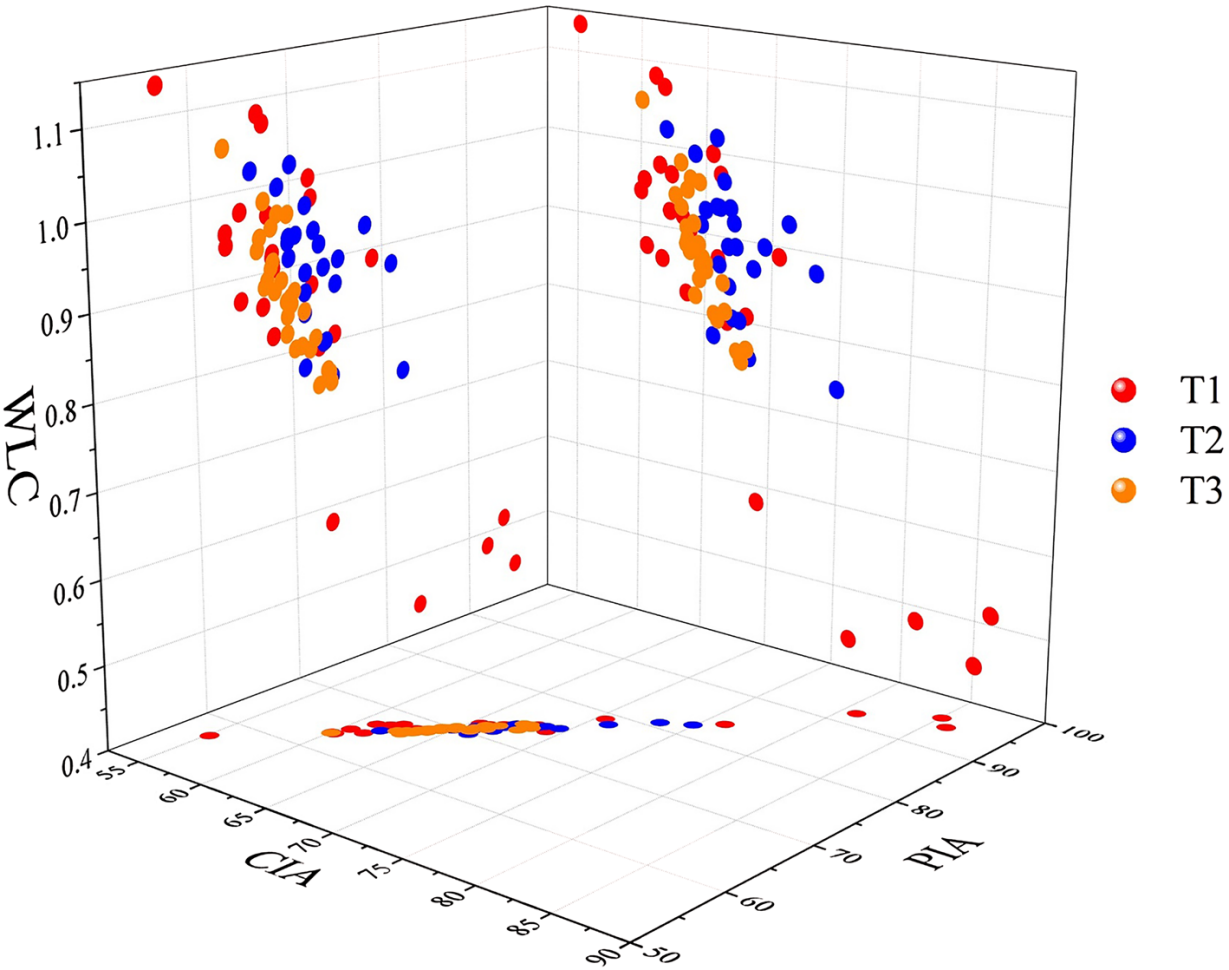
Distribution and correlation of the chemical weathering index of soil profiles undergoing different pedogenic processes. CIA = chemical index of alteration; WLC = weathering leaching coefficient; PIA = plagioclase index of alteration.

The CIA followed the order of T2 (65.78–68.64) > T1 (64.81–67.97) > T3 (63.49–66.12); the PIA followed the order of T2 (70.75–74.80) > T1 (70.08–74.44) > T3 (67.78–71.55) (Table 2). The greater the PIA value, the higher the weathering degree of plagioclase. This indicates that compared with NE, TP accelerated the weathering of plagioclase, while EP reduced the weathering. T2 was at the stage of moderate chemical weathering, while T1 and T3 are at the transition stage from weak weathering intensity to moderate chemical weathering. However, to amend the CIA value, Nesbitt and Young et al.^31^ proposed the A-CN-K ternary diagram, which not only reflects the trend of chemical weathering but also reflects the change in mineral composition during the process of chemical weathering. Figure 6 shows that the chemical weathering trend parallels the CN–A boundary, which reflects that plagioclase is weakly resistant to weathering, resulting in rapid leaching of Ca and Na, while potassium feldspar, which is rich in K, is relatively stable. The mineral composition is mainly dominated by illite and montmorillonite and has not reached the level of kaolinite dominance. Additionally, the WLC is an effective index to reflect soil chemical weathering intensity. We analysed the correlations of the CIA, PIA and WLC and found that there was a significant positive correlation between CIA and PIA (*r* = 0.992**, *P* < 0.01), a significant negative correlation between CIA and WLC (*r* = − 0.807**, *P* < 0.01) and a significant negative correlation between PIA and WLC (*r* = − 0.796**, *P* < 0.01) (Fig. 7). In summary, the chemical weathering intensity of the tested soil was weak. The CIA and PIA yielded similar results that reflect the intensity of soil chemical weathering with the order of T2 > T1 > T3.

## Discussion

### Anthropic activities change the degree and direction of soil development

Anthropic activities, as a key factor of soil mixing, mainly alter pedogenesis by indirectly influencing five soil-forming factors, among which tillage is the most common anthropic activity affecting pedogenesis^32^. However, with the rapid development of agriculture, traditional farming modes have been unable to meet the current development of agriculture, society and economy, and farming plot reconstruction engineering may become an important measure to promote the rapid development of agriculture today^15, 33^. Therefore, it is very important to explore the influence of natural evolution and anthropic activities on pedogenesis and the differences in soil genetic characteristics under the influence of two kinds of anthropic activities (TP and EP). Generally, weathering indices are also used to evaluate the degree of soil development, and the higher the weathering degree is, the higher the degree of soil development^34^. Aaron et al.^35^ showed a highly significant positive linear trend between the soil development indices and CIA at CIA values greater than 53%. However, in this study, compared with T1, T2 had a lower profile development degree, while T3 had a higher profile development degree (Fig. 3). The chemical weathering intensity of T1, T2 and T3 followed the order of T2 > T1 > T3 (Table 2). This finding indicates that the influence of anthropic activities on soil genesis and evolution is complicated. On the one hand, anthropic activities (EPs) enhance soil development but decrease the soil chemical weathering intensity. On the other hand, anthropic activities (TPs) promote soil chemical weathering, but weaken soil development. These differences may be attributed to the influence of soil erosion. The soil in the study area is characterized by a shallow soil horizon, high rock fragment content, low soil organic matter content and poor water stability of aggregates^36, 37^. Before farming plot reconstruction engineering, the soil genetic characteristics of the study area were mainly influenced by tillage perturbation. Although tillage can accelerate soil-forming speed^7^, the high degree of land use and unreasonable farming methods easily increase the risk of soil erosion^38^. Moreover, the unique topographical conditions of hilly mountainous regions are likely to cause the risk of soil erosion under the conditions of high-intensity rainfall^39^. Soil erosion causes a series of problems, such as shallow soil horizons, poor soil structure and low clay contents, and weakens the degree of soil profile development^40, 41^. With increasing soil erosion intensity, the soil may undergo intense chemical weathering^42, 43^.

Furthermore, farming plot reconstruction engineering has been applied to the farmland to improve agricultural production capacity and optimize land structure^38^. The core of farming plot reconstruction engineering is to reduce the slope of the original sloping farmland by terracing the sloping field and stripping and backfilling the topsoil, which can effectively control soil erosion and improve soil physical and chemical properties^5, 44^. In engineering construction, to ensure the thickness of cultivated soil (50 cm) after engineering, it is necessary to excavate and backfill earthwork^16^. In the process of excavating and backfilling earthwork, the thickness of the soil horizon of the fill earthwork area more easily meets the design requirement of 50 cm than that of the excavation earthwork area and has better physical and chemical properties. In contrast, if the thickness of the soil horizon of the excavation earthwork area cannot meet the design requirement of 50 cm, it is necessary to excavate and transport the parent material to accelerate the rapid weathering of the parent material. Strong engineering perturbations cause rock fragments to be brought into cultivated soil, which increases the rock fragment content in the soil and shows corresponding weak chemical weathering characteristics. Therefore, farming plot reconstruction engineering strongly affects the soil-forming process; on the one hand, they enhance soil development, and on the other hand, the soil after engineering reconstruction contains a large amount of rock fragments, which changes the direction of soil development.

### Highlight the research on soil classification considering anthropic activities

To understand, utilize and transform nature, people have long studied and classified natural bodies in their environment. As for soil, with the development of soil science, mastering the theoretical basis and methods of soil classification is essential for us to understand various soil classification systems and to then develop and perfect soil classification systems. At present, many countries worldwide have carried out relevant research on soil classification. The most representative systems are the Soil Taxonomy (ST) and World Reference Base (WRB) systems^22, 45^. The WRB not only incorporates some concepts and terms of soil genetic classification but is also based on the diagnostic layer and diagnostic characteristics of the ST and highlights its own characteristics^46^. With the deepening of human understanding of soil classification and the increasingly serious influence of anthropic activities (cultivation, irrigation, fertilization, addition of exogenous substances and engineering perturbation, etc.) on the soil-forming process, the WRB has established two RSGs: Anthrosols and Technosols^22^. However, at present, there are no Anthrosol orders in ST. Therefore, the WRB has made outstanding contributions to unified soil classification worldwide.

At present, in the WRB, Anthrosols mainly include Hydragric Anthrosols, Irragric Anthrosols, Hortic Anthrosols, Plaggic Anthrosols, Pretic Anthrosols and Terric Anthrosols^22^. According to the diagnostic layer and diagnostic characteristics of the WRB, the soils do not meet the standard of Anthrosols after farming plot reconstruction engineering. At the same time, in the WRB, two diagnostic materials are defined for Technosols: artefacts and technic hard material^22^. Artefacts are mainly substances created or greatly changed by human beings or brought from depth to the surface, where they are not affected by surface processes, while technic hard materials are consolidated materials derived from an industrial process^47^. In this study, the engineering perturbation process involves only topsoil stripping and backfilling and parent material excavation and transport, which indicates that the soils do not meet the Technosol standards after farming plot reconstruction engineering. The results in the present paper are basically the same as those in Schad^47^, and human-transported natural soil does not belong to Technosols. Therefore, although the WRB has established the RSGs of Anthrosols and Technosols, the soil after farming plot reconstruction engineering is neither an Anthrosol nor a Technosol. This shows that scholars have performed limited research on anthropogenic soils, especially soil, after engineering reconstruction. In the future, we should pay much more attention to the soil disturbed by subjectively anthropic activities and gradually shift the research of soil classification considering the anthropic activities and their related indices. Moreover, this study can provide a new idea for the continuous improvement of soil classification systems in the future.

## Conclusion

This study proves that the influence of anthropic activities (tillage perturbation and engineering perturbation) on soil-forming characteristics is complex. On the one hand, engineering perturbations enhance soil development, but decrease the soil chemical weathering intensity. On the other hand, tillage perturbations promote soil chemical weathering, but weaken soil development. Although anthropic activities can reduce the bulk density, clay content, silt content, soil organic carbon, total nitrogen, pH and cation exchange capacity, these physical and chemical characteristics are improved and with a lower variable coefficient after engineering perturbation. Anthropic activities have greatly affected the formation and evolution of soil, especially the impact of farming plot reconstruction engineering on pedogenesis. This influence not only changes the configuration of the soil profile (soil profile patterns from A–C to A–B–C) but also increases the soil layer thickness. The ownership of engineering disturbed soil in the Soil Taxonomy and World Reference Base cannot be found only according to diagnostic layer and diagnostic characteristics. Therefore, to adapt to the development of current agricultural intensification, we should change the research focus of soil genetic classification from naturally synergetic processes to subjectively anthropic activities.

## Supplementary Information

Supplementary Information.
