# Supplementary material for: Changes in the profile properties and chemical weathering characteristics of cultivated soils affected by anthropic activities
Source: Sci Rep. 2021 Oct 21;11:20822. doi: 10.1038/s41598-021-00302-w (PMC8531366; doi:10.1038/s41598-021-00302-w)
Supplement: Supplementary file 1 — Supplementary Information. [file 41598_2021_302_MOESM1_ESM.docx]

Appendix A. Quantification of profile descriptions and calculation of morphological properties.

| Morphological properties | Assignment and calculation formula | | | | |
| --- | --- | --- | --- | --- | --- |
| Rubification | Assignment /Hue | | | | |
|  | 10/5Y←9/2.5Y←8/10YR←7/7.5YR←6/5YR←5/2.5YR←4/10R←3/7.5R←2/5R←1/2.5R | | | | |
|  | Assignment /Chroma | | | | |
|  | 8/1←7/2←6/3←5/4←4/5←3/6←2/7←1/8 | | | | |
|  | Rubification=10×(Hue+Chroma)_(dry)_+10(Hue+Chroma)_(moist)_ | | | | |
| Standardization | *X*_sr_=*X*_r_/360 | | | | |
|  |  | | | | |
| Melanization | Assignment /Value | | | | |
|  | 8/1←7/2←6/3←5/4←4/5←3/6←2/7←1/8 | | | | |
|  | Melanization=10×(Value_(dry)_+Value_(moist)_) | | | | |
| Standardization | *X*_sm_=*X*_m_/160 | | | | |
|  |  | | | | |
| Total texture | Assigned from 1 to 12 crossing toward clay on textural triangle/Texture | | | | |
|  | Assignment /Cohersiveness | | | | |
|  | 1/Not cohersive→2/Slightly cohersive→3/Cohersive→4/Very cohersive | | | | |
|  | Assignment /Plasticity | | | | |
|  | 1/Not plastic→2/Slightly plastic→3/Plastic→4/Very plastic | | | | |
|  | Total texture=10×(Texture+Cohersiveness+Plasticity) | | | | |
| Standardization | *X*_st_=*X*_t_/200 | | | | |
|  |  | | | | |
| Structure | Quantification | 5 | 10 | 20 | 30 |
|  | Grade |  | s | m | w |
|  | Type | pl | gr | pr | col |
|  |  |  | sbk |  |  |
|  |  |  | abk |  |  |
|  | Structure=(grade+type) | | | | |
| Standardization | *X*_ss_=*X*_s_/60 | | | | |
|  |  | | | | |
| Moist consistence | Assignment /Consistence | | | | |
|  | 1/Loose→2/Slightly loose→3/Slightly firm→4/Firm→5/Very firm→6/Extremely firm | | | | |
|  | Moist consistence=10×moist consistence | | | | |
| Standardization | *X*_sc_=*X_m_*_c_/60 | | | | |
| Horizion index(HI) | *HI*=(*X*_sr_+*X*_sm_+*X*_st_+*X*_ss_+*X*_sc_)/5 | | | | |
| PDI | $PDI=\sum(HI\times\text{d）}$ | | | | |

Note: PDI= profile development index; s=strong; m=moderate; w=weak; pl=platy; gr=granular; pr=prismatic; col=columnar; sbk=subangular blocky; abk= angular blocky; d=horizion depth (cm); *X*_r_, *X*_m_, *X*_t_, *X*_s_ and *X*_mc_ is the assignment of every kinds of morphological properties.

Appendix B. Description of soil profile macromorphological characteristics in different pedogenic processes.

| Sample types |  | Profile no |  | Horizon |  | Depth |  | Soil colour |  |  |  | Soil structure |  | Texture |  | Cohesiveness |  | Plasticity |  | Moist consistence |
| --- | --- | --- | --- | --- | --- | --- | --- | --- | --- | --- | --- | --- | --- | --- | --- | --- | --- | --- | --- | --- |
|  |  |  |  |  |  | (cm) |  | Wet state |  | Dry state |  |  |  |  |  |  |  |  |  |  |
| T1 |  | S1 |  | A |  | 0-18 |  | 5YR4/3 |  | 5YR4/4 |  | gr |  | lc |  | C |  | p |  | L |
|  |  |  |  | B |  | 18-40 |  | 5YR5/3 |  | 5YR4/3 |  | pr |  | scl |  | NC |  | np |  | F |
|  |  |  |  | C |  | > 40 |  | 5YR5/3 |  | 5YR5/3 |  |  |  |  |  |  |  |  |  |  |
|  |  | S2 |  | A |  | 0-24 |  | 5YR4/3 |  | 5YR4/4 |  | abk |  | c |  | VC |  | vp |  | SF |
|  |  |  |  | B |  | 24-33 |  | 5YR5/3 |  | 5YR4/3 |  | abk |  | c |  | VC |  | vp |  | F |
|  |  |  |  | C |  | > 33 |  | 5YR5/3 |  | 5YR5/3 |  |  |  |  |  |  |  |  |  |  |
|  |  | S3 |  | A |  | 0-33 |  | 5YR5/3 |  | 5YR4/3 |  | gr |  | lc |  | C |  | p |  | SF |
|  |  |  |  | B |  | 33-59 |  | 5YR5/3 |  | 5YR5/3 |  | abk |  | lc |  | C |  | p |  | SF |
|  |  |  |  | C |  | > 59 |  | 5YR6/3 |  | 5YR6/3 |  |  |  |  |  |  |  |  |  |  |
|  |  | S4 |  | A |  | 0-25 |  | 5YR4/3 |  | 5YR4/4 |  | abk |  | scl |  | NC |  | np |  | L |
|  |  |  |  | C |  | > 25 |  | 5YR5/3 |  | 5YR4/3 |  |  |  |  |  |  |  |  |  |  |
|  |  | S5 |  | A |  | 0-21 |  | 5YR4/4 |  | 5YR4/5 |  | abk |  | lc |  | C |  | p |  | L |
|  |  |  |  | C |  | > 21 |  | 5YR5/4 |  | 5YR4/4 |  |  |  |  |  |  |  |  |  |  |
|  |  | S6 |  | A |  | 0-23 |  | 5YR4/4 |  | 5YR4/4 |  | gr |  | lc |  | SC |  | sp |  | L |
|  |  |  |  | B |  | 23-53 |  | 5YR5/4 |  | 5YR4/5 |  | abk |  | lc |  | SC |  | sp |  | SL |
|  |  |  |  | C |  | > 53 |  | 5YR5/4 |  | 5YR5/4 |  |  |  |  |  |  |  |  |  |  |
|  |  | S7 |  | A |  | 0-16 |  | 5YR4/4 |  | 5YR4/4 |  | gr |  | c |  | VC |  | vp |  | L |
|  |  |  |  | B |  | 16-30 |  | 5YR5/4 |  | 5YR5/4 |  | abk |  | c |  | VC |  | vp |  | SF |
|  |  |  |  | C |  | > 30 |  | 5YR5/4 |  | 5YR5/4 |  |  |  |  |  |  |  |  |  |  |
|  |  | S8 |  | A |  | 0-18 |  | 5YR4/4 |  | 5YR4/5 |  | gr |  | lc |  | C |  | p |  | L |
|  |  |  |  | C |  | > 18 |  | 5YR5/4 |  | 5YR5/4 |  |  |  |  |  |  |  |  |  |  |
|  |  | S9 |  | A |  | 0-20 |  | 5YR4/4 |  | 5YR4/4 |  | gr |  | lc |  | C |  | p |  | SF |
|  |  |  |  | C |  | > 20 |  | 5YR5/4 |  | 5YR5/4 |  |  |  |  |  |  |  |  |  |  |
|  |  | S10 |  | A |  | 0-24 |  | 5YR4/4 |  | 5YR4/4 |  | abk |  | cl |  | SC |  | sp |  | L |
|  |  |  |  | C |  | > 24 |  | 5YR5/4 |  | 5YR4/4 |  |  |  |  |  |  |  |  |  |  |
| T2 |  | P1 |  | A |  | 0-15 |  | 5YR6/3 |  | 5YR5/3 |  | abk |  | cl |  | SC |  | sp |  | SL |
|  |  |  |  | C |  | >15 |  | 5YR6/2 |  | 5YR5/2 |  |  |  |  |  |  |  |  |  |  |
|  |  | P2 |  | A |  | 0-22 |  | 5YR6/3 |  | 5YR5/3 |  | gr |  | cl |  | SC |  | sp |  | L |
|  |  |  |  | B |  | 22-40 |  | 5YR6/3 |  | 5YR5/3 |  | abk |  | l |  | NC |  | np |  | SL |
|  |  |  |  | C |  | > 40 |  | 5YR6/2 |  | 5YR5/2 |  |  |  |  |  |  |  |  |  |  |
|  |  | P3 |  | A |  | 0-20 |  | 5YR5/3 |  | 5YR5/3 |  | gr |  | lc |  | C |  | p |  | L |
|  |  |  |  | B |  | 20-40 |  | 5YR6/3 |  | 5YR5/3 |  | abk |  | cl |  | SC |  | sp |  | SL |
|  |  |  |  | C |  | > 40 |  | 5YR6/2 |  | 5YR6/2 |  |  |  |  |  |  |  |  |  |  |
|  |  | P4 |  | A |  | 0-15 |  | 5YR5/3 |  | 5YR4/3 |  | abk |  | lc |  | C |  | p |  | SF |
|  |  |  |  | C |  | > 15 |  | 5YR6/2 |  | 5YR5/3 |  |  |  |  |  |  |  |  |  |  |
|  |  | P5 |  | A |  | 0-22 |  | 5YR6/2 |  | 5YR6/3 |  | abk |  | scl |  | SC |  | sp |  | L |
|  |  |  |  | C |  | > 22 |  | 5YR6/2 |  | 5YR6/2 |  |  |  |  |  |  |  |  |  |  |
|  |  | P6 |  | A |  | 0-25 |  | 5YR6/2 |  | 5YR5/2 |  | gr |  | sc |  | NC |  | np |  | L |
|  |  |  |  | C |  | > 25 |  | 5YR6/2 |  | 5YR6/2 |  |  |  |  |  |  |  |  |  |  |
|  |  | P7 |  | A |  | 0-33 |  | 5YR5/2 |  | 5YR5/3 |  | gr |  | sc |  | NC |  | np |  | SL |
|  |  |  |  | C |  | > 33 |  | 5YR6/2 |  | 5YR6/2 |  |  |  |  |  |  |  |  |  |  |
|  |  | P8 |  | A |  | 0-20 |  | 5YR5/3 |  | 5YR5/3 |  | abk |  | scl |  | SC |  | sp |  | L |
|  |  |  |  | C |  | > 20 |  | 5YR6/2 |  | 5YR6/2 |  |  |  |  |  |  |  |  |  |  |
|  |  | P9 |  | A |  | 0-25 |  | 5YR6/3 |  | 5YR5/3 |  | gr |  | sl |  | NC |  | np |  | L |
|  |  |  |  | C |  | > 25 |  | 5YR6/2 |  | 5YR6/3 |  |  |  |  |  |  |  |  |  |  |
|  |  | P10 |  | A |  | 0-15 |  | 5YR6/3 |  | 5YR5/3 |  | gr |  | sl |  | NC |  | np |  | L |
|  |  |  |  | B |  | 15-55 |  | 5YR6/3 |  | 5YR6/3 |  | abk |  | sl |  | NC |  | np |  | SL |
|  |  |  |  | C |  | > 55 |  | 5YR6/2 |  | 5YR6/2 |  |  |  |  |  |  |  |  |  |  |
| T3 |  | P′1 |  | A |  | 0-30 |  | 5YR6/3 |  | 5YR5/3 |  | gr |  | cl |  | SC |  | sp |  | L |
|  |  |  |  | C |  | > 30 |  | 5YR6/2 |  | 5YR5/2 |  |  |  |  |  |  |  |  |  |  |
|  |  | P′2 |  | A |  | 0-20 |  | 5YR5/3 |  | 5YR5/3 |  | gr |  | lc |  | C |  | p |  | L |
|  |  |  |  | B |  | 20-60 |  | 5YR6/3 |  | 5YR5/3 |  | abk |  | cl |  | SC |  | sp |  | SL |
|  |  |  |  | C |  | > 60 |  | 5YR6/2 |  | 5YR5/2 |  |  |  |  |  |  |  |  |  |  |
|  |  | P′3 |  | A |  | 0-25 |  | 5YR5/3 |  | 5YR4/3 |  | abk |  | cl |  | SC |  | sp |  | L |
|  |  |  |  | B |  | 25-60 |  | 5YR6/2 |  | 5YR5/2 |  | abk |  | cl |  | SC |  | sp |  | L |
|  |  |  |  | C |  | > 60 |  | 5YR6/2 |  | 5YR6/2 |  |  |  |  |  |  |  |  |  |  |
|  |  | P′4 |  | A |  | 0-30 |  | 5YR5/3 |  | 5YR4/3 |  | abk |  | lc |  | C |  | p |  | L |
|  |  |  |  | C |  | > 30 |  | 5YR6/2 |  | 5YR5/3 |  |  |  |  |  |  |  |  |  |  |
|  |  | P′5 |  | A |  | 0-20 |  | 5YR5/3 |  | 5YR5/3 |  | abk |  | cl |  | SC |  | sp |  | L |
|  |  |  |  | B |  | 20-53 |  | 5YR5/2 |  | 5YR5/3 |  | abk |  | sl |  | NC |  | np |  | L |
|  |  |  |  | C |  | > 53 |  | 5YR6/2 |  | 5YR5/3 |  |  |  |  |  |  |  |  |  |  |
|  |  | P′6 |  | A |  | 0-22 |  | 5YR5/3 |  | 5YR5/3 |  | gr |  | cl |  | SC |  | sp |  | L |
|  |  |  |  | B |  | 22-40 |  | 5YR6/2 |  | 5YR5/2 |  | abk |  | cl |  | SC |  | sp |  | L |
|  |  |  |  | C |  | > 40 |  | 5YR6/2 |  | 5YR6/2 |  |  |  |  |  |  |  |  |  |  |
|  |  | P′7 |  | A |  | 0-25 |  | 5YR5/2 |  | 5YR5/3 |  | gr |  | cl |  | SC |  | sp |  | L |
|  |  |  |  | B |  | 25-40 |  | 5YR6/2 |  | 5YR6/2 |  | abk |  | cl |  | SC |  | sp |  | L |
|  |  |  |  | C |  | > 40 |  | 5YR6/2 |  | 5YR6/2 |  |  |  |  |  |  |  |  |  |  |
|  |  | P′8 |  | A |  | 0-20 |  | 5YR5/3 |  | 5YR5/3 |  | abk |  | cl |  | SC |  | sp |  | L |
|  |  |  |  | B |  | 20-50 |  | 5YR6/3 |  | 5YR5/3 |  | abk |  | l |  | NC |  | np |  | L |
|  |  |  |  | C |  | > 50 |  | 5YR6/2 |  | 5YR6/2 |  |  |  |  |  |  |  |  |  |  |
|  |  | P′9 |  | A |  | 0-25 |  | 5YR5/3 |  | 5YR4/3 |  | gr |  | cl |  | SC |  | sp |  | L |
|  |  |  |  | B |  | 25-50 |  | 5YR6/3 |  | 5YR5/3 |  | abk |  | sl |  | NC |  | np |  | L |
|  |  |  |  | C |  | > 50 |  | 5YR6/2 |  | 5YR6/3 |  |  |  |  |  |  |  |  |  |  |
|  |  | P′10 |  | A |  | 0-25 |  | 5YR5/3 |  | 5YR4/3 |  | gr |  | lc |  | C |  | p |  | L |
|  |  |  |  | B |  | 25-55 |  | 5YR5/2 |  | 5YR5/3 |  | abk |  | l |  | NC |  | np |  | L |
|  |  |  |  | C |  | > 55 |  | 5YR6/2 |  | 5YR6/2 |  |  |  |  |  |  |  |  |  |  |

Note: T1, T2 and T3 represent the soils undergoing natural evolution (NE), tillage perturbation (TP) and engineering perturbation (EP); gr= granular; abk= angular blocky; pr= prismatic; lc=loam clay; c=clay; cl=clay loam; sl=sandy loam; l=loam; sc=sandy clay; scl=sandy clay loam; C= Cohesive; NC=Not cohesive; SC= Slightly cohesive; VC=Very cohesive; p= plastic; np= not plastic; sp=slightly plastic; vp=very plastic; L=Loose; SL= Slightly loose; SF=Slightly firm; F=Firm.
